# Propofol-Based Anesthesia Maintenance and/or Volatile Anesthetics during Intracranial Aneurysm Repair: A Comparative Analysis of Neurological Outcomes

**DOI:** 10.3390/jcm12216954

**Published:** 2023-11-06

**Authors:** Shooka Esmaeeli, Negar Motayagheni, Andres Brenes Bastos, Christopher S Ogilvy, Ajith J Thomas, Richard Pollard, Lauren K Buhl, Maxwell B Baker, Sheshanna Phan, Omron Hassan, Corey R Fehnel, Matthias Eikermann, Shahzad Shaefi, Ala Nozari

**Affiliations:** 1 Department of Anesthesiology, Critical Care and Pain Medicine, Beth Israel Deaconess Medical Center, Harvard Medical School, Boston, MA 02215, USA; rpollard@bidmc.harvard.edu (R.P.); sshaefi@bidmc.harvard.edu (S.S.); 2 Department of Anesthesiology, Boston Medical Center, Boston University, Boston, MA 02118, USA; shooka.esmaeeli@bmc.org (S.E.); maxb98@bu.edu (M.B.B.); 3 Heart Transplant Program, Cedars-Sinai California Heart Center, Beverly Hills, CA 90211, USA; negar.motayagheni@csmns.org; 4 Department of Anesthesiology, Yale New Haven Hospital, Yale University School of Medicine, New Haven, CT 06510, USA; andres.brenesbastos@yale.edu; 5 Division of Neurosurgery, Beth Israel Deaconess Medical Center, Harvard Medical School, Boston, MA 02215, USA; cogilvy@bidmc.harvard.edu; 6 Department of Neurosurgery, Cooper University Hospital, Cooper Medical School of Rowan University, Camden, NJ 08103, USA; thomas-ajith@cooperhealth.edu; 7 Department of Anesthesiology, Dartmouth Hitchcock Medical Center, Dartmouth Geisel School of Medicine, Hanover, NH 03766, USA; 8 Department of Internal Medicine, University of New Mexico Hospital, University of New Mexico School of Medicine, Albuquerque, NM 87106, USA; sphan@salud.unm.edu; 9 Department of Internal Medicine, Freeman Hospital, Joplin, MO 64804, USA; 10 Department of Neurology, Beth Israel Deaconess Medical Center, Harvard Medical School, Boston, MA 02215, USA; 11 Department of Anesthesiology, Critical Care, Pain Medicine, Montefiore Medical Center, Albert Einstein College of Medicine, Bronx, NY 10467, USA; meikermann@montefiore.org

**Keywords:** unruptured intracranial aneurysm, general anesthesia, propofol, volatile agents, endovascular surgery, craniotomy

## Abstract

Background: Volatile and intravenous anesthetics have substantial effects on physiological functions, notably influencing neurological function and susceptibility to injury. Despite the importance of the anesthetic approach, data on its relative risks or benefits during surgical clipping or endovascular treatments for unruptured intracranial aneurysms (UIAs) remains scant. We investigated whether using volatile anesthetics alone or in combination with propofol infusion yields superior neurological outcomes following UIA obliteration. Methods: We retrospectively reviewed 1001 patients who underwent open or endovascular treatment for UIA, of whom 596 had short- and long-term neurological outcome data (modified Rankin Scale) recorded. Multivariable ordinal regression analysis was performed to examine the association between the anesthetic approach and outcomes. Results: Of 1001 patients, 765 received volatile anesthetics alone, while 236 received propofol infusion and volatile anesthetics (combined anesthetic group). Short-term neurological outcome data were available for 619 patients and long-term data for 596. No significant correlation was found between the anesthetic approach and neurologic outcomes, irrespective of the type of procedure (open craniotomy or endovascular treatment). The combined anesthetic group had a higher rate of ICU admission (*p* < 0.001) and longer ICU and hospital length of stay (LOS, *p* < 0.001). Similarly, a subgroup analysis revealed longer ICU and hospital LOS (*p* < 0.0001 and *p* < 0.001, respectively) in patients who underwent endovascular UIA obliteration under a combined anesthetic approach (n = 678). Conclusions: The addition of propofol to volatile anesthetics during UIA obliteration does not provide short- or long-term benefits to neurologic outcomes. Compared to volatile anesthetics alone, the combination of propofol and volatile anesthetics may be associated with an increased rate of ICU admission, as well as longer ICU and hospital LOS.

## 1. Introduction

With a prevalence of approximately 3–5% and an annual rupture risk of 0.95%, unruptured intracranial aneurysms (UIAs) remain a major cause of hemorrhagic stroke with significant morbidity and mortality [1,2,3,4]. Novel endovascular techniques provide an increasing array of treatment options for complex aneurysms, while surgical innovations, such as retractorless surgery, minimally invasive craniotomies, and novel diagnostic and reconstructive techniques, continue to improve patient safety and outcomes [5]. Although rare, hemorrhagic or ischemic injuries from aneurysmal perforation, cerebral hypoperfusion, or thromboembolism remain among the most devastating complications [6]. It is critical to minimize the risk of these complications and mitigate neurologic injury by optimizing surgical conditions through careful hemodynamic and respiratory control, as well as the optimization of fluid and metabolic balance, in order to achieve cellular homeostasis [7,8,9]. Therefore, anesthetic neuroprotection can play an important role in preserving neurologic function and improving outcomes, particularly in patients at risk for ischemic or hemorrhagic complications [10].

Volatile and intravenous anesthetics have major neurological and cardiovascular effects that have been studied and documented extensively in, among others types of studiy, experimental and clinical studies of ischemic stroke. These anesthetics are reported to possess neuroprotective properties that may decrease the risk of neurological injury [11,12]. In particular, propofol is suggested to induce neuroprotection through a wide range of physiological effects including its antioxidant activity, ability to reduce cerebral metabolism, and effects on GABAergic neuronal activity and excitotoxicity [13,14]. As a result, it has been hypothesized that a “multimodal” anesthetic regimen using propofol infusion could lead to better neurologic outcomes in patients at risk for perioperative stroke [13]. A recent retrospective study of 314,932 non-cardiac cases, however, documented a dose-dependent protective effect of volatile anesthetics, but not propofol, on the incidence and severity of postoperative ischemic stroke [15]. Further, in a retrospective study of 84 patients who underwent endovascular management of acute ischemic stroke, the best neurological outcomes were observed when volatile agents were used compared to total intravenous anesthesia (TIVA) with propofol or a combination thereof [16].

Even though previous studies have provided general guidelines for the anesthetic management of cerebral aneurysm surgery [7,17,18], there is currently only one systematic review exploring the risks and benefits of common anesthetic agents during UIA treatment [10]. A recent study also reported neuroprotective effects of propofol post-conditioning in patients undergoing the open repair of intracranial aneurysms [19], but there are currently no studies assessing the long-term neurologic outcomes with different anesthetic regimens for UIA treatment. Accordingly, we aimed to investigate the outcome effects of anesthesia maintenance with propofol or volatile anesthetics during UIA obliteration. We hypothesized that the anesthetic choice does not affect the overall or neurologic outcomes following UIA surgery.

## 2. Materials and Methods

### 2.1. Study Population

**Figure 1:**
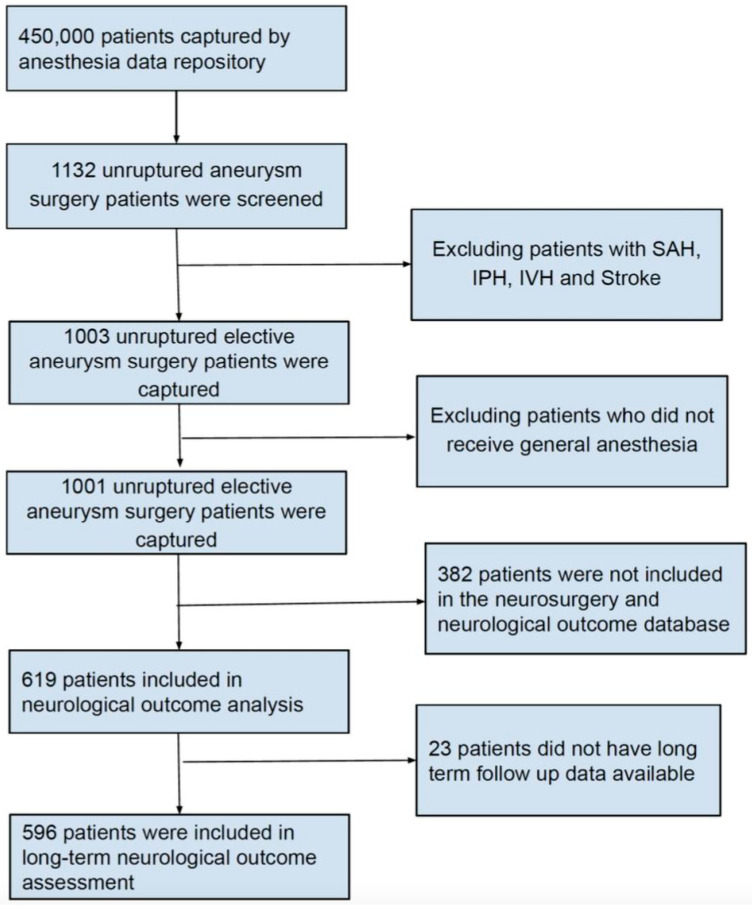
Flow chart showing the study inclusion criteria.

After obtaining study approval from the Institutional Review Board, we retrospectively examined data from all patients who underwent endovascular or open treatment for UIA between January 2014 and December 2018 at the Beth Israel Deaconess Medical Center (BIDMC). All data originated from the Anesthesia Research Data Repository and the Neurosurgery Outcome Database for aneurysm surgery at BIDMC. The need for patient consent was waived due to the nature of the study. The Anesthesia Research Data Repository included demographic characteristics as well as intraoperative data, such as administered drug doses, blood pressure, fluid volumes, and blood product transfusion. The Neurosurgery Outcome Database for aneurysm surgery included the type of surgery and the Modified Rankin Scale for Neurologic Disability (mRS), documented during neurosurgical evaluations before and repeatedly after surgery. Only patients with unruptured intracranial aneurysms were included. Patient data were merged from these two sources to create a single de-identified database of all patients with UIA treated at BIDMC, excluding those who had a documented history of aneurysm rupture and SAH (Figure 1).

We further divided our study population into two groups based on the anesthetic agents used: the volatile anesthetic group, which included patients who received maintenance anesthesia only with volatile anesthetic agents, and the combined anesthetic group, which included patients who received maintenance anesthesia with both volatile anesthetic agents and propofol. Two patients received total intravenous anesthesia (TIVA) with propofol without volatile anesthetics, and were therefore excluded from further analysis (Figure 1).

### 2.2. Outcome Measures

The primary outcome measures were short-term and long-term neurologic outcomes, determined using the mRS, calculated during the first 30 days after UIA treatment and the last follow up neurosurgery visit (between 6–12 months), as documented in each patient’s electronic medical record. Patients were categorized into neurologically intact (mRS = 0), good neurologic outcome (mRS = 1), and neurologic disability (mRS = 2–5) groups. There was no mortality (mRS = 6) among our study population. The secondary outcomes were intensive care unit admission and length of stay (ICU-LOS) and hospital length of stay (H-LOS).

### 2.3. Statistical Analysis

Continuous data are reported as means ± standard deviation or medians with interquartile ranges (IQR) and were compared between groups using *t*-tests or Wilcoxon signed-rank tests, as indicated. Categorical data are expressed as proportions and were compared using chi-squared tests.

Multivariable ordinal regression analysis was performed to examine the association between the anesthetic approach and outcome, while controlling for the following biologically relevant covariates: (1) age; (2) sex; (3) American Society of Anesthesiologists physical (ASA) status; (4) body mass index (BMI). Age and BMI were considered continuous variables, while sex (female versus male) and propofol infusion (yes or no), were considered dichotomous variables. The dosage of volatile anesthetics was documented using the average age-adjusted Minimum Alveolar Concentration (MAC), and opioid dosage as the Oral Morphine Equivalent of opioid (OME) during the perioperative period. All analyses were conducted using R (The R Foundation for Statistical Computing, Vienna, Austria) [20]. Odds ratios (ORs) are reported with 95% confidence intervals (CIs). *p*-values of 0.05 or less and 95% CIs that did not cross one were considered to be significant.

## 3. Results

A total of 450,000 patients underwent procedures under general anesthesia at BIDMC during the study period, of which 1132 underwent endovascular or open repair of UIAs. After applying the exclusion criteria, 1001 patients who underwent elective UIA surgery were included in the study. Short-term neurologic assessments were documented for 619 of the final cohort, and 596 patients were followed up for an average duration of 5 months (IQR 1–12 months) and had documented long-term neurologic assessments (Figure 1).

Anesthetic maintenance was performed with volatile anesthetics alone in 765 patients (volatile anesthetic group), and propofol infusion plus volatile anesthetics in 236 (combined anesthetic group). The demographic and clinical data, stratified by anesthetic group, are summarized in Table 1. ASA physical status was comparable between the two groups (*p* = 0.1). Patients in the combined anesthetic group were younger (57 ± 11 vs. 59 ± 12, *p* = 0.01) and more likely to have undergone an open craniotomy (78% in combined group vs. 19% in volatile anesthetic group, *p* < 0.001). The aneurysm morphology was documented in 616 patients, 380 (62%) of whom underwent endovascular and 236 (38%) open surgery. In the endovascular group, the mean aneurysm size measured 8.6 ± 0.6 mm, while in the open surgery group, it was notably smaller at 5.0 ± 0.3 mm (*p* < 0.001). The majority of the aneurysms fell within the size range of 5–9.9 mm, accounting for 44% of the cases. Approximately 27% of the aneurysms were smaller, measuring < 4.9 mm, while 17% were in the 10–14.9 mm range, and 12% were larger, measuring ≥ 15 mm. The majority of aneurysms, constituting 94% of the cases, exhibited a saccular morphology, while the remaining 6% were fusiform in shape. Geographically, 90% of these aneurysms were distributed within the anterior circulation, primarily localized in the middle cerebral artery, anterior communicating artery, and the C6 segment of the internal carotid artery.

The demographic and clinical data, stratified by treatment approach, are shown in Table 2. ASA physical status was comparable between the two groups (*p* = 0.8). With an average age of 56 ± 11 years, patients who underwent craniotomy were younger than those requiring an endovascular approach (60 ± 12, *p* < 0.0001). Propofol infusion was used more often among open craniotomy procedures compared to endovascular procedures (*p* < 0.001).

**Figure 2:**
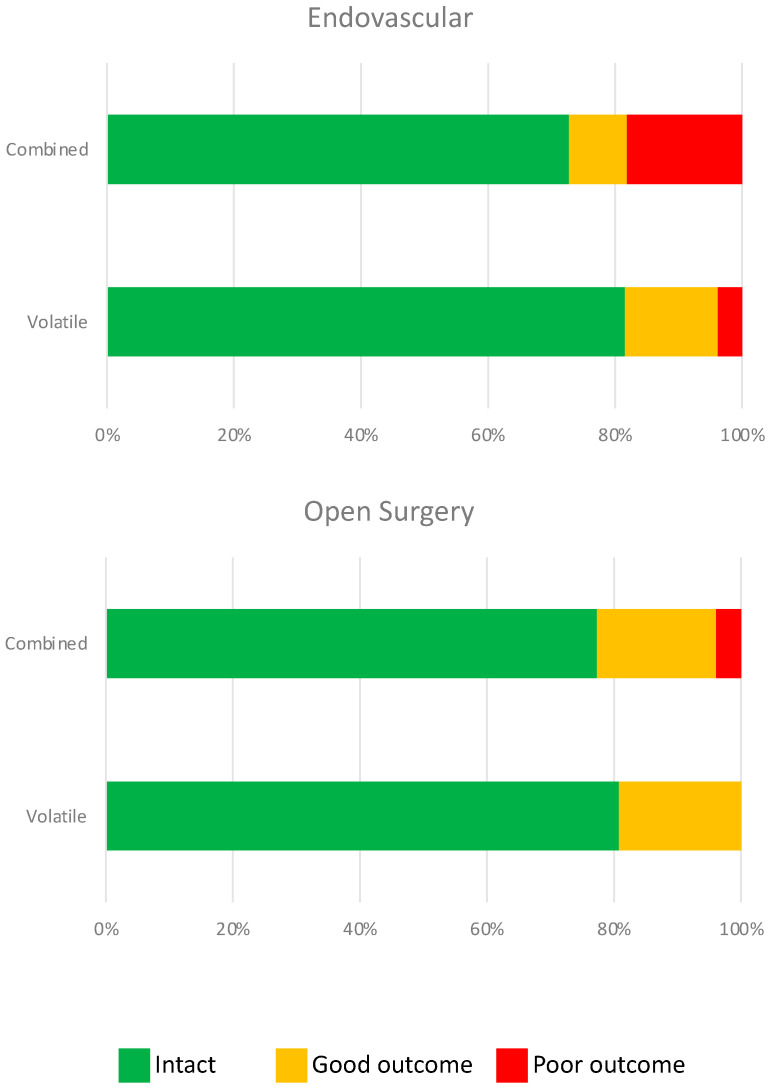
Neurological outcome was not statistically different after endovascular (*p* = 0.20) or open surgery (*p* = 0.44) with combined or volatile anesthetics. Green = neurologically intact (mRS = 0), orange = good neurologic outcome (mRS = 1), and red = poor neurologic outcome (mRS = 2–5).

The baseline and long-term neurologic outcomes are summarized in Table 3. There were no significant differences in the baseline or long-term neurologic outcomes between groups. Multivariable ordinal regression analysis revealed no significant correlation between the maintenance anesthetic used and long-term neurologic outcomes, irrespective of the surgical approach (endovascular vs. open; Table 4 and Figure 2). We did, however, find a significant correlation between age (*p* = 0.002), BMI (*p* = 0.005), and long-term neurologic outcome.

Secondary outcome variables are summarized in Table 5. With regard to surgical approach, the rate of ICU admission was lower (*p* < 0.01), and ICU-LOS (*p* < 0.001) and H-LOS (*p* < 0.001) were shorter following endovascular treatment of UIA compared to open craniotomy. With regard to maintenance anesthetics, a higher rate of ICU admission (*p* < 0.001), longer ICU-LOS (*p* < 0.001), and longer H-LOS (*p* < 0.001) were observed in the combined anesthetic group. A subgroup analysis based on treatment approach (endovascular vs. open craniotomy) showed comparable patterns. Among patients who underwent endovascular obliteration, H-LOS (*p* < 0.0001) and ICU-LOS (*p* < 0.001) were longer after combined maintenance anesthetics. However, the ICU admission rate was not statistically different (76% vs. 67%) in the combined anesthetic group. Among patients who underwent open craniotomy, ICU-LOS (2.8 ± 3.7 vs. 2.3 ± 3.4 days) and H-LOS (5.2 ± 4.6 vs. 4.6 ± 3.7) were not significantly different between the two anesthetic groups. However, UIA patients had a higher ICU admission rate (81% versus 69%, *p* < 0.01) after open craniotomy and clipping when a combined anesthetic approach was elected.

## 4. Discussion

In this cohort of 1001 patients following UIA treatment, 596 with long-term neurologic outcome data, the addition of a propofol infusion to volatile anesthetics was not associated with improved outcomes. Surprisingly, the combined propofol and volatile anesthetic approach was associated with a higher rate of ICU admission and prolonged ICU and hospital LOS compared to volatile anesthetics alone, which raises concerns about potential adverse neurophysiological consequences.

The precise mechanism underlying the association between propofol infusion and an increased rate of ICU admission and length of stay (ICU-LOS) remains elusive. However, several factors might contribute to this phenomenon, including potential hemodynamic, neurovascular, or metabolic effects, or even a complex pharmacodynamic interaction between propofol and volatile anesthetics. It is also important to highlight that a substantial proportion of patients (78%) who received combined anesthetic maintenance underwent open craniotomy procedures. Notably, these open craniotomies were associated with considerably longer procedure times compared to the endovascular group (averaging 209 min versus 122 min). Given this discrepancy in surgical duration, it could be argued that the observed variations in secondary outcomes may be primarily attributed to these surgical factors, with limited direct influence from the chosen anesthetic approach. It is, therefore, essential to consider the potential confounding effects of the surgical procedure itself, which could be a significant contributor to the observed differences in patient outcomes.

As is summarized in Table 3, however, the neurological outcome did not differ between the endovascular and open surgery groups, nor was the anesthetic approach associated with outcome differences in either of these subgroups. Accordingly, these findings support the main conclusion that the neurological outcome is not influenced by the above approaches to anesthesia maintenance during UIA repair. One could also contend that the potential neuroprotective effects of propofol might be offset by the necessity to lower the concentration of inhaled volatile anesthetics. This hypothesis aligns with recent findings by Raub et al., who demonstrated that volatile anesthetics exhibit a dose-dependent neuroprotective effect, thereby diminishing the occurrence and severity of perioperative ischemic strokes [15].

Although both propofol and inhalational anesthetics are commonly used for the maintenance of anesthesia, they are often used as the sole anesthetic agent for most surgical procedures, and not in combination. There is currently a notable absence of conclusive evidence or established guidelines pertaining to the optimal anesthetic approach for the repair of UIA. Consequently, some institutions have formulated their own individual protocols, while others delegate the decision to the discretion of individual anesthetists. One promising approach involves the synergistic use of propofol in combination with volatile agents. This combination strategy appears to offer several advantages, including the potential reduction in total drug dosage requirements for each individual agent. This reduction, in turn, may mitigate the risk of associated complications such as cerebral vasodilation and intracranial pressure (ICP) effects, metabolic side-effects, and the potential development of propofol infusion syndrome. Furthermore, the propofol–volatile agent combination has been suggested to potentially decrease the incidence of postoperative nausea and vomiting (PONV), and may be associated with a shorter time to extubation, reduced intraoperative movement by the patient, and a more favorable postoperative perception of pain [21]. An additional argument in favor of this combined approach is its potential to provide enhanced neuroprotection for patients who are expected to experience neuronal injury during the procedure. Moreover, it facilitates the feasibility of intraoperative electrophysiological monitoring, offering a multifaceted approach to improving patient outcomes during UIA repair [22,23,24]. Propofol has anti-inflammatory properties and may protect against cell damage caused by hypoxia-induced oxidative stress [13,25]. Moreover, propofol provides neuroprotection by decreasing the cerebral metabolic requirement for oxygen (CMRO_2_) while maintaining cerebrovascular reactivity to carbon dioxide [26]. Unlike inhalational anesthetics, propofol does not cause direct cerebral vasodilation and can therefore provide better intraoperative brain relaxation [13,26,27]. Accordingly, propofol infusion is more commonly considered during open craniotomies as opposed to the endovascular treatment of UIA.

Despite a growing body of evidence of the neuroprotective properties of propofol, clinical studies have failed to show a significant benefit to patient outcomes compared to inhaled anesthetics. The incidence of postoperative complications is similar when using propofol vs. volatile anesthetics during elective craniotomies [28]. It has also been suggested that volatile anesthetics are more effective than propofol in mitigating cortical spreading depolarization, a common complication of subarachnoid bleeding that can complicate postoperative neurologic recovery [29,30]. Yet, randomized studies report better cognitive outcomes with volatile anesthetics compared to TIVA with propofol for carotid endarterectomy and cardiac surgery [31,32], and volatile anesthetics have also been reported to better protect against ischemic stroke after non-cardiac surgery, with a dose-dependent effect on both the incidence and severity of the stroke [15].

Our study differs from earlier reports in that we examined a combined maintenance anesthetic approach with propofol in addition to volatile anesthetics, which could provide information on the combined neuroprotective effects of these commonly used anesthetic agents. Given that both treatment groups in our study received volatile anesthetics, the absence of a difference in neurologic outcomes between groups leads us to reject the hypothesis that the addition of a propofol infusion provides superimposed neuroprotection. Moreover, the increased rate of ICU admission and ICU-LOS we observed in the combined anesthetic group is concerning in terms of the negative physiological effects of this combination, although it could also be related to other confounders that were not controlled for in our retrospective study.

One such confounder is the difference in the proportion of patients who received propofol infusion during endovascular treatment vs. open surgery (8% and 57%, respectively). Given that the ICU admission rate, ICU-LOS, and H-LOS were higher after open craniotomy, it could be argued that the observed increase in ICU admission rate, ICU-LOS, and H-LOS in the combined anesthetic group is attributable to the type of surgery and the preoperative group differences that determined the surgical approach (e.g., aneurysm size, type, and location and patient comorbidities) or physiologic differences between open and endovascular UIA treatment. Subgroup analysis, however, revealed a similar trend, with prolonged ICU-LOS and H-LOS in the combined anesthetic group for patients undergoing endovascular repair of UIA, as well as a higher ICU admission rate after open craniotomies. These findings can therefore signal important neurological effects of maintenance anesthetics, specifically the addition of propofol to volatile anesthetics, irrespective of the surgical approach. Moreover, preoperative comorbidities and ASA physical status did not differ between the combined and volatile anesthetic groups. Thus, while the inherent complexity of open craniotomy for aneurysm clipping could contribute to the observed differences in secondary outcomes, it is likely not the only cause of a prolonged ICU-LOS and H-LOS.

When considering study limitations, our study, like other retrospective studies, did not randomly assign patients to an anesthetic regimen. Provider bias and other uncontrolled confounders could certainly have affected the outcome. Moreover, we did not examine the effects of propofol infusion alone (without inhaled anesthetics) given the small number of these patients (n = 2), precluding the assessment of patient outcomes following TIVA in this population. Lastly, the mRS score used as our primary outcome measure is a relatively crude estimate of neurologic outcome, and although it is an established metric with excellent inter- and intra-rater reliability, it has a relatively low clinical sensitivity and does not measure more nuanced differences in cognitive performance [33].

The endovascular and open treatment of UIA are associated with significant cerebrovascular and systemic physiological perturbations and require an anesthetic approach that optimizes cerebral hemodynamics and maximizes perioperative neuroprotection. Despite the theoretical advantages of combining volatile anesthetics with propofol for anesthetic maintenance, our data support the use of only volatile anesthetics for both endovascular and open UIA treatment and showed no benefit of the addition of a propofol infusion. Although the observed association of propofol with ICU-LOS and H-LOS does not indicate causation, it is concerning in terms of the potential adverse effects of the combined anesthetics.

Our study demonstrates that the addition of propofol to volatile anesthetics does not provide any long-term or short-term benefits to neurologic outcomes among patients undergoing treatment for UIA. The combination of propofol and volatile anesthetics also showed worse secondary outcomes, including ICU admission rates, ICU-LOS, and H-LOS, compared to volatile anesthetics alone. The use of a combined propofol–volatile maintenance anesthetic should be carefully considered in this patient population as it might result in unanticipated adverse events, including prolonged ICU and hospital LOS. Further investigation with prospective, randomized studies is warranted to gain a better understanding of how the addition of propofol to a volatile maintenance anesthetic affects patient outcomes following UIA treatment.

## Figures and Tables

**Table 1:** Demographic and clinical features of the study cohort based on the anesthetic agent groups.

(Total N = 1001)	Combined * (N = 236)	Inhalational † (N = 765)	*p* Value
Age (years)	57 ± 11	59 ± 12	0.01
Sex			
Female	170 (72%)	587 (76%)	0.1
Male	66 (28%)	178 (24%)	
BMI	28 ± 6	27± 5	0.3
ASA Status			
I	0 (0%)	11 (1%)	0.1
II	76 (26%)	257 (33%)	
III	141 (65%)	467 (61%)	
IV	18 (6%)	30 (4%)	
V	1 (0.4%)	0 (0%)	
Anesthetic agents			
N_2_O	17 (8%)	58 (7%)	0.9
Sevoflurane	215 (91%)	695 (90%)	1
Desflurane	28 (11%)	82 (10%)	0.7
Isoflurane	10 (5%)	13 (1%)	0.04
Opioids	230 (97%)	756 (99%)	0.2
Treatment			
Endovascular management	53 (22%)	625 (81%)	<0.001
Open craniotomy	183 (78%)	140 (19%)	
Duration of procedure (min)	210 ± 10	162 ± 3	<0.001

* Combined group = patients who received both propofol infusion and inhalational agents; † inhalational anesthetic group = patients who received inhalation anesthetics without propofol infusion. Data are presented as mean ± standard deviation, or proportions, and were compared using *t*-tests, Kruskal–Wallis tests, and chi-squared tests, respectively.

**Table 2:** Demographics, clinical parameters, and anesthetic approaches based on the type of surgical intervention.

(Total N = 1001)	Endovascular * (N = 678)	† Open Craniotomy (N = 323)	*p* Value
Age (years)	60 ± 12	56 ± 11	<0.0001
Sex			
Female	520 (77%)	237 (73%)	0.2
Male	158 (23%)	86 (27%)	
BMI	28 ± 6	27 ± 5	0.1
ASA Status			
I	10 (1%)	1 (0.5%)	0.8
II	223 (33%)	110 (34%)	
III	413 (61%)	195 (60%)	
IV	32 (5%)	16 (5%)	
V	0 (0%)	1 (0.5%)	
Anesthetic agents			
N_2_O	52 (8%)	23 (7%)	0.86
Sevoflurane	614 (90%)	296 (92%)	0.66
Desflurane	68 (11%)	42 (13%)	0.19
Isoflurane	2 (1%)	21 (7%)	<0.001
Opioids	664 (97%)	322 (99%)	0.06
Propofol infusion	53 (8%)	183 (57%)	<0.001
Duration of procedure (min)	122 ± 6	209 ± 5	<0.001

* Endovascular group = patients who underwent endovascular obliteration of intracranial aneurysm; † open craniotomy group = patients who underwent open craniotomy. Data are presented as mean ± standard deviation, or proportions, and were compared using *t*-tests, Kruskal–Wallis tests, and chi-squared tests, respectively.

**Table 3:** (a) Neurological outcomes after repair of unruptured intracranial aneurysms in different anesthetic and treatment groups; (b) neurological outcomes in patients with open versus endovascular approach to intracranial aneurysm repair.

(a)							
	Baseline mRS (Total N = 619)				Long-term mRS (Total N = 596)		
	Neurologically intact	Good neurologic outcome	Poor neurologic outcome		Neurologically intact	Good neurologic outcome	Poor neurologic outcome
Volatile (N = 466)	289 (62%)	150 (32%)	27 (6%)	Volatile (N = 446)	363 (81%)	70 (16%)	13 (2%)
Combined (N = 153)	100 (65%)	46 (30%)	7 (5%)	Combined (N = 150)	115 (77%)	26 (17%)	4 (3%)
*p* value	0.4			*p* value	0.1		
(b)							
	Baseline mRS (Total N = 619)				Long-term mRS (Total N = 596)		
	Neurologically intact	Good neurologic outcome	Poor neurologic outcome		Neurologically intact	Good neurologic outcome	Poor neurologic outcome
Endovascular (N = 382)	235 (62%)	119 (31%)	28 (7%)	Endovascular (N = 364)	295 (81%)	52 (14%)	17 (5%)
Open (N = 237)	154 (65%)	77 (32%)	6 (3%)	Open (N = 232)	183 (79%)	44 (19%)	5 (2%)
*p* value	0.2			*p* value	0.6		

Patients were categorized into neurologically intact (mRS = 0), good neurologic outcome (mRS = 1), and poor neurologic outcome (mRS = 2–5) groups. There was no mortality (mRS = 6) among the patients. Data are presented as proportions, and were compared using Kruskal–Wallis test.

**Table 4:** Multivariable ordinal regression analysis to examine the association between the anesthetic approach and long-term neurologic outcome.

Variables	Coefficients	Odds Ratio	95% CI	*p* Value
N_2_O	0.11 (0.39)	1.13	0.72–1.13	0.7
Sevoflurane	−0.33 (0.56)	0.72	0.22–2.18	0.5
Desflurane	−0.25 (0.48)	0.77	0.28–1.90	0.5
Isoflurane	0.61 (1.20)	1.84	0.08–16.6	0.6
Propofol infusion	0.29 (0.23)	1.33	0.83–2.10	0.1
Opioids	−0.31 (1.13)	0.72	0.10–14.6	0.7
Age	−0.02 (0.008)	0.97	0.95–0.99	0.002
Sex	−0.18 (0.25)	0.83	0.49–1.36	0.4
ASA status	0.22 (0.20)	1.23	0.83–1.85	0.2
BMI	0.04 (0.01)	1.04	1.01–1.08	0.005

Standard errors are reported in parentheses.

**Table 5:** Secondary outcome variables of unruptured intracranial aneurysm management in different groups.

	ICU Admission	ICU-LOS		H-LOS	
Anesthetic (N) ± SD					
Volatile (765)	67%	2 (0–2)	2.1 ± 3.5	2 (2–3)	3.8 ± 5.6
Combined (236)	80%	2 (2–3)	3.5 ± 5.4	4 (3–6)	5.7 ± 6
*p* value	<0.001	<0.001		<0.001	
Treatment (N)					
Endovascular (678)	68%	2 (0–2)	2.4 ± 4.3	2 (2–3)	3.9 ± 6.4
Open Craniotomy (323)	76%	2 (2–3)	2.7 ± 3.6	4 (3–5)	4.9 ± 4.2
*p* value	<0.01	<0.001		<0.001	
EndovascularManagement (N)					
Volatile (625)	67%	2 (0–2)	2.1 ± 3.6	2 (2–3)	3.6 ± 5.9
Combined (53)	76%	2 (2–4)	5.9 ± 8.8	3 (2–11)	7.6 ± 9.2
*p* value	0.2	<0.001		<0.001	
Open Craniotomy					
Volatile (140)	69%	2 (0–3)	2.3 ± 3.4	3 (3–5)	4.6 ± 3.7
Combined (183)	81%	2 (2–3)	2.8 ± 3.7	4 (3–5)	5.2 ± 4.6
*p* value	0.01	0.08		0.2	

The ICU admission rate was higher and length of stay (LOS) longer in the combined anesthesia group, along with an extended hospital LOS. Considering that similar trends were observed in patients undergoing open craniotomy versus the endovascular approach, we conducted a separate comparison of anesthetic choices within each surgical group. In the open craniotomy group, ICU admission rates remained elevated with combined anesthesia, while in the endovascular group, this anesthetic approach was associated with prolonged ICU-LOS and hospital LOS (by almost 4 days). Data are presented as medians (interquartile ranges), means (±standard deviation), or proportions, and were compared using Wilcoxon signed-rank test and chi-squared test, respectively.

## Data Availability

Data access is restricted due to privacy and ethical considerations.

## Abbreviation List

ASA	American Association of Anesthesiologists
BIDMC	Beth Israel Deaconess Medical Center
BMI	Body mass index
CI	Confidence interval
H-LOS	Hospital length of stay
ICU	Intensive care unit
ICU-LOS	Intensive care unit length of stay
IQR	Interquartile range
LOS	Length of stay
MAC	Minimum Alveolar Concentration
mRS	Modified Rankin Scale
OME	Oral Morphine Equivalents
OR	Odds ratio
SAH	Subarachnoid hemorrhage
TIVA	Total intravenous anesthesia
UIA	Unruptured intracranial aneurysm

## References

[1] Chalouhi N., Hoh B.L., Hasan D. (2013). Review of cerebral aneurysm formation, growth, and rupture. Stroke.

[2] Benjamin E.J., Blaha M.J., Chiuve S.E., Cushman M., Das S.R., Deo R., De Ferranti S.D., Floyd J., Fornage M., Gillespie C. (2017). Heart Disease and Stroke Statistics-2017 Update: A Report from the American Heart Association. Circulation.

[3] Kochanek K.D., Murphy S.L., Xu J., Arias E. (2013). Mortality in the United States, 2013. NCHS Data Brief..

[4] Go A.S., Mozaffarian D., Roger V.L., Benjamin E.J., Berry J.D., Borden W.B., Bravata D.M., Dai S., Ford E.S., Fox C.S. (2013). Heart Disease and Stroke Statistics—2013 Update: A report from the American Heart Association. Circulation.

[5] Kalani M.Y.S., Wanebo J.E., Martirosyan N.L., Nakaji P., Zabramski J.M., Spetzler R.F. (2017). A raised bar for aneurysm surgery in the endovascular era. J. Neurosurg..

[6] Ihn Y.K., Shin S.H., Baik S.K., Choi I.S. (2018). Complications of endovascular treatment for intracranial aneurysms: Management and prevention. Interv. Neuroradiol..

[7] Bruder N., Boussen S., Velly L. (2019). Anesthesia for Aneurysmal Subarachnoid Hemorrhage. Textbook of Neuroanesthesia and Neurocritical Care.

[8] Hunt W.E., Hess R.M. (1968). Surgical Risk as Related to Time of Intervention in the Repair of Intracranial Aneurysms. J. Neurosurg..

[9] Molyneux A.J., Kerr R.S., Yu L.-M., Clarke M., Sneade M., Yarnold J.A., Sandercock P. (2005). International subarachnoid aneurysm trial (ISAT) of neurosurgical clipping versus endovascular coiling in 2143 patients with ruptured intracranial aneurysms: A randomised comparison of effects on survival, dependency, seizures, rebleeding, subgroups, and aneurysm occlusion. Lancet.

[10] Esmaeeli S., Valencia J., Buhl L.K., Bastos A.B., Goudarzi S., Eikermann M., Fehnel C., Pollard R., Thomas A., Ogilvy C.S. (2021). Anesthetic management of unruptured intracranial aneurysms: A qualitative systematic review. Neurosurg. Rev..

[11] Archer D.P., Walker A.M., McCann S.K., Moser J.J., Appireddy R.M. (2017). Anesthetic Neuroprotection in Experimental Stroke in Rodents: A Systematic Review and Meta-analysis. Anesthesiology.

[12] Wang H., Li P., Xu N., Zhu L., Cai M., Yu W., Gao Y. (2016). Paradigms and mechanisms of inhalational anesthetics mediated neuroprotection against cerebral ischemic stroke. Med. Gas Res..

[13] Adembri C., Venturi L., Pellegrini-Giampietro D.E. (2007). Neuroprotective effects of propofol in acute cerebral injury. CNS Drug Rev..

[14] Bayona N.A., Gelb A.W., Jiang Z., Wilson J.X., Urquhart B.L., Cechetto D.F. (2004). Propofol neuroprotection in cerebral ischemia and its effects on low-molecular-weight antioxidants and skilled motor tasks. Anesthesiology.

[15] Raub D., Platzbecker K., Grabitz S.D., Xu X., Wongtangman K., Pham S.B., Murugappan K.R., Hanafy K.A., Nozari A., Houle T.T. (2021). Effects of Volatile Anesthetics on Postoperative Ischemic Stroke Incidence. J. Am. Heart Assoc..

[16] Sivasankar C., Stiefel M., Miano T.A., Kositratna G., Yandrawatthana S., Hurst R., Kofke W.A. (2016). Anesthetic variation and potential impact of anesthetics used during endovascular management of acute ischemic stroke. J. Neurointerv. Surg..

[17] Lecours M., Gelb A.W. (2015). Anesthesia for the surgical treatment of cerebral aneurysms. Colomb. J. Anesthesiol..

[18] Chowdhury T., Petropolis A., Wilkinson M., Schaller B., Sandu N., Cappellani R.B. (2014). Controversies in the anesthetic management of intraoperative rupture of intracranial aneurysm. Anesthesiol. Res. Pract..

[19] Guo D., Li Y., Wang H., Wang X., Hua W., Tang Q., Miao L., Wang G. (2019). Propofol post-conditioning after temporary clipping reverses oxidative stress in aneurysm surgery. Int. J. Neurosci..

[20] R Core Team (2018). R: A Language and Environment for Statistical Computing.

[21] Wolf A., Selpien H., Haberl H., Unterberg M. (2021). Does a combined intravenous-volatile anesthesia offer advantages compared to an intravenous or volatile anesthesia alone: A systematic review and meta-analysis. BMC Anesthesiol..

[22] Higashida R.T., Lahue B.J., Torbey M.T., Hopkins L.N., Leip E., Hanley D.F. (2007). Treatment of unruptured intracranial aneurysms: A nationwide assessment of effectiveness. AJNR Am. J. Neuroradiol..

[23] Badenes R., Nato C.G., Peña J.D., Bilotta F. (2021). Inhaled anesthesia in neurosurgery: Still a role?. Best Pract. Res. Clin. Anaesthesiol..

[24] Bilotta F., Guerra C., Rosa G. (2013). Update on anesthesia for craniotomy. Curr. Opin. Anaesthesiol..

[25] Chen R.M., Chen T.G., Chen T.L., Lin L.L., Chang C.C., Chang H.C., Wu C.H. (2005). Anti-inflammatory and antioxidative effects of propofol on lipopolysaccharide-activated macrophages. Ann. N. Y. Acad. Sci..

[26] Oshima T., Karasawa F., Satoh T. (2002). Effects of propofol on cerebral blood flow and the metabolic rate of oxygen in humans. Acta Anaesthesiol. Scand..

[27] Kaisti K.K., Metsähonkala L., Teräs M., Oikonen V., Aalto S., Jääskeläinen S., Hinkka S., Scheinin H. (2002). Effects of Surgical Levels of Propofol and Sevoflurane Anesthesia on Cerebral Blood Flow in Healthy Subjects Studied with Positron Emission Tomography. Anesthesiology.

[28] Chui J., Mariappan R., Mehta J., Manninen P., Venkatraghavan L. (2014). Comparison of propofol and volatile agents for maintenance of anesthesia during elective craniotomy procedures: Systematic review and meta-analysis. Can. J. Anaesth..

[29] Kitahara Y., Taga K., Abe H., Shimoji K. (2001). The effects of anesthetics on cortical spreading depression elicitation and c-fos expression in rats. J. Neurosurg. Anesthesiol..

[30] Chung D.Y., Oka F., Ayata C. (2016). Spreading Depolarizations: A Therapeutic Target Against Delayed Cerebral Ischemia After Subarachnoid Hemorrhage. J. Clin. Neurophysiol..

[31] Kuzkov V.V., Obraztsov M.Y., Ivashchenko O.Y., Ivashchenko N.Y., Gorenkov V.M., Kirov M.Y. (2018). Total Intravenous Versus Volatile Induction and Maintenance of Anesthesia in Elective Carotid Endarterectomy: Effects on Cerebral Oxygenation and Cognitive Functions. J. Cardiothorac. Vasc. Anesth..

[32] Schoen J., Husemann L., Tiemeyer C., Lueloh A., Sedemund-Adib B., Berger K.U., Hueppe M., Heringlake M. (2011). Cognitive function after sevoflurane- vs propofol-based anaesthesia for on-pump cardiac surgery: A randomized controlled trial. Br. J. Anaesth..

[33] Banks J.L., Marotta C.A. (2007). Outcomes validity and reliability of the modified Rankin scale: Implications for stroke clinical trials: A literature review and synthesis. Stroke.

